# A scoping review on family medicine in sub-Saharan Africa: practice, positioning and impact in African health care systems

**DOI:** 10.1186/s12960-020-0455-4

**Published:** 2020-04-03

**Authors:** Maaike Flinkenflögel, Vincent Sethlare, Vincent Kalumire Cubaka, Mpundu Makasa, Abraham Guyse, Jan De Maeseneer

**Affiliations:** 1 Health Unit, KIT Royal Tropical Institute, Amsterdam, the Netherlands; 2 Department of Primary Health Care, School of Medicine and Pharmacy, College of Medicine and Health Sciences, University of Rwanda, Kigali, Rwanda; 3 Department of Public Health and Primary Care, Ghent University, Ghent, Belgium; 4 Primafamed-Network, Cape Town, South Africa; 5 Department of Family Medicine and Public Health Medicine, University of Botswana, Gaborone, Botswana; 6 Department of Research and Training, Partners In Health, Inshuti Mu Buzima, Kigali, Rwanda; 7 Department of Primary Health Care, School of Medicine and Pharmacy, College of Medicine and Health Sciences, University of Rwanda, Kigali, Rwanda; 8 Centre for Global Health, Department of Public Health, Aarhus University, Aarhus, Denmark; 9 Department of Community and Family Medicine, School of Public Health, University of Zambia, Lusaka, Zambia; 10 Department of Family Medicine, University of Calabar Teaching Hospital, Calabar, Nigeria

**Keywords:** Family Medicine, Family physician, Primary Health Care, Africa, Health systems strengthening

## Abstract

**Background:**

Family medicine (FM) is a relatively new discipline in sub-Saharan Africa (SSA), still struggling to find its place in the African health systems. The aim of this review was to describe the current status of FM in SSA and to map existing evidence of its strengths, weaknesses, effectiveness and impact, and to identify knowledge gaps.

**Methods:**

A scoping review was conducted by systematically searching a wide variety of databases to map the existing evidence. Articles exploring FM as a concept/philosophy, a discipline, and clinical practice in SSA, published in peer-reviewed journals from 2000 onwards and in English language, were included. Included articles were entered in a matrix and then analysed for themes. Findings were presented and validated at a Primafamed network meeting, Gauteng 2018.

**Results:**

A total of 73 articles matching the criteria were included. FM was first established in South Africa and Nigeria, followed by Ghana, several East African countries and more recently additional Southern African countries. In 2009, the Rustenburg statement of consensus described FM in SSA. Implementation of the discipline and the roles and responsibilities of family physicians (FPs) varied between and within countries depending on the needs in the health system structure and the local situation. Most FPs were deployed in district hospitals and levels of the health system, other than primary care. The positioning of FPs in SSA health systems is probably due to their scarcity and the broader mal-distribution of physicians. Strengths such as being an “all- round specialist”, providing mentorship and supervision, as well as weaknesses such as unclear responsibilities and positioning in the health system were identified. Several studies showed positive perceptions of the impact of FM, although only a few health impact studies were done, with mixed results.

**Conclusions:**

FM is a developing discipline in SSA. Stronger evidence on the impact of FM on the health of populations requires a critical mass of FPs and shared clarity of their position in the health system. As FM continues to grow in SSA, we suggest improved government support so that its added value and impact on health systems in terms of health equity and universal health coverage can be meaningfully explored.

## Background

Family medicine (FM) is a clinical discipline, with family physicians (FPs) at its core, focused on primary health care (PHC), which provides care to individuals and communities. The discipline aims to strengthen health systems (HS) in order to achieve health equity and universal health coverage (UHC), and to leave no one behind in pursuing the Sustainable Development Goals (SDGs) [1–3]. Uncertainties for policymakers and other stakeholders relate to what role this discipline may have, where it should be placed in the HS, and what benefits it could offer. FM is relatively new in many sub-Saharan African (SSA) countries, and discussions on its definition, roles, positioning, practice, and impact are ongoing [4–8].

Defining FM has proven to be complex and varies in different settings around the globe. However, some principles are common to FM worldwide: relevance, accessibility, improved equity, comprehensiveness, person-centred care, cost-effectiveness, quality care, scientific and contextual evidence, integration in PHC, coordination of care, sustainability and innovation (Table 1) [9, 10]. Although its fundamental principles are described similarly, the practice can be quite different when comparing FM in SSA and high-income countries (HIC) [6–8, 11, 12].

**Table 1:** WONCA (World Organisation of Family Doctors) Definition of family physician (1991) [9]

“The physician who is primarily responsible for providing first contact and comprehensive health care to every individual seeking medical care and advice, and arranging for other health personnel to provide services as necessary. The family physician functions as a generalist who accepts everyone seeking care in contrast to other physicians who limit access to their services on the basis of age, sex and/or type of health problem.”	

While FPs are the first point of contact in many HIC, in SSA this is primarily the responsibility of nurses and community health workers, due to the low density of physicians [13, 14]. FPs in SSA are mainly working at other levels in the health system (secondary/tertiary) and are often based in district hospitals where there is insufficient availability of other specialists [7]. At the same time, they have responsibilities for the whole district, such as supervising primary care facilities and addressing broader public health issues [7]. Such contextual differences shape the scope of practice and the required competencies of FPs. In several SSA countries, FPs have a clinical leadership and governance role in district health and in PHC teams [13, 15–17]. Different countries use different terminology related to FM, which is clarified in Table 2.

**Table 2:** Specific terminology related to the discipline and human resources in family medicine in sub-Saharan Africa ([7, 8, 38], anecdotal)

*Medical doctors (MDs)* without further specialization have received different terms in the African setting, such as medical officer in South Africa, general practitioner in Rwanda, medical doctor in Kenya. Wherever needed, we decided to refer to doctors without further training as MDs. *General practitioners (GPs)* - in Nigeria are seen as family physicians (with further specialization), similar as in Europe - in South Africa are seen as primary care doctors working in the private sector without further specialization - in Rwanda are seen as medical doctors without further specialization working in hospitals *Due to this variety in terminology, we have not used the term GP, unless further explained in text.* *Family physicians (FPs)* are medical doctors with 2 to 4 years postgraduate training within the specialization of family medicine and registered as a specialist in the specific country. The *Discipline of Family Medicine* could apply to more than one type of health professional (for example in SA family medicine also trains clinical associates and clinical nurse practitioners). Though in this article, we use the term *discipline of FM* as the medical specialty that deploys family physicians. *General (Medical) Practice* was the name of the postgraduate discipline in West African countries such as Nigeria and Ghana at start of the programme. In the early 2000s this was changed to family medicine.	

In 2017, according to order of inception, FM was operating in South Africa, Nigeria, Uganda, Democratic Republic of Congo (DRC), Sudan, Ghana, Tanzania, Kenya, Lesotho, Botswana, Somaliland, Ethiopia, Mali and Malawi [4]^1^. The development of the discipline was strengthened by several initiatives, such as the Primafamed (Primary health care and family medicine education)-Network and the World Organisation of Family Doctors (Wonca) in Africa, with the learning community being a strong asset [18, 19]. There exists no standardization of training programmes between countries, even though there is a strong need for accreditation and quality assurance [18] The African Journal of Primary Health Care and Family Medicine has provided an academic platform since 2008 while it contributes to “a contextual and holistic view of family medicine and primary health care as practised across the continent” [20]

At a global level, there is evidence that FM is cost-effective and delivers good health outcomes, at low cost, with high user satisfaction [3, 21, 22]. Dr. Chan, a previous director-general of the World Health Organization (WHO), described FM as “our highest hope for the future” [23] In SSA, however, a lack of clarity on the scope and practice of FM among policymakers often leads to the discipline not being fully integrated into health systems.

This scoping review started by looking at the definition of FM, its adaptation to HS in SSA and its potential role in supporting PHC, as defined by WHO in the Astana Declaration [24]. The aim of this review was to describe the current status of FM in SSA and to provide an overview of existing evidence of its strengths, weaknesses, effectiveness and impact. Identification of knowledge gaps should inform the development of a research strategy to provide additional evidence needed by policymakers in SSA as they strive to achieve the SDGs. To our knowledge, at the time of this review, no such scoping or systematic review of the evidence for FM in SSA was published.

## Methods

The scoping review was conducted to address the research question: “what is the contribution of FM in strengthening health systems in sub-Saharan Africa”. Additional sub-questions are listed in Table 3. This scoping review was performed as part of a larger project to identify the global priorities for PHC research and to establish a new global research consortium. In SSA, the Primafamed network performed this scoping review and two others that addressed community-oriented primary care and measurement of PHC systems [25, 26].

**Table 3:** Sub-questions of the literature review on the contribution of Family Medicine in Africa health systems

1. What are the different ways in which FM has been implemented in Africa? 2. What evidence exists for the effectiveness and impact of FM in Africa? 3. What is known about the strengths and weaknesses of FM as part of health systems in Africa? 4. Where are family physicians deployed in African health systems? 5. What roles do family physicians play in African Health systems?	

### The search strategy

The scoping review protocol was conducted according to a pre-determined protocol [27]. Medical subject heading (MeSH) terms and search strings were agreed upon (see Table 4) [27, 28]. The databases searched in March and April 2018 are shown in Table 5.

**Table 4:** Search strings and key words/ MeSH terms used in the search [22]

1. Family Practice/organization & administration (OG) OR Family Practice/ education (ED) AND Africa 2. Physicians AND (community health services OR primary health care) AND Africa 3. Primary Care Physicians AND delivery of health care/organization & administration (OG) AND Africa 4. Family Practice AND (Health Care Quality, Access, and Evaluation OR cost-benefit analysis) AND Africa 5. (Family Physicians OR Family Practice) AND delivery of health care/organization & administration (OG) AND Africa

**Table 5:** Databases used for the literature review

• Systematic reviews: Cochrane library, Epistemonikos, Trip databases • General databases: PubMed/ Medline and Google Scholar • African databases: Sabinet online, Africa wide information, African journals online	

Inclusion criteria for articles identified in the search strategies are shown in Table 6. Selected articles had to meet criteria 1 to 3, and optional criteria 4 or 5 as shown in Table 6. All types of articles from peer-reviewed journals were considered, but grey literature was not searched. Only articles published in English since the year 2000 were included. Publications before 2000 were few and less relevant to current health systems. Research articles that looked at delivery of care for specific diseases were excluded.

**Table 6:** Inclusion criteria for the articles in this review. Criteria 1, 2 and 3 were obligatory for inclusion, and criteria 4 and 5 were optional

Criteria for the articles	Explanation
1. Physician/medical doctor with a postgraduate degree in FM	The article should focus on delivery of care by doctors/physicians who have received a postgraduate degree in FM
2. Implementation	The article should focus on implementation of FM (role and place in the health system)
3. Sub-Saharan Africa	The article should focus on FM in at least one SSA country
4. Effectiveness/impact	The article should focus on effectiveness/impact (perceived value, cost-effectiveness) of FM
5. Strengths and weaknesses	The article should focus on positive and negative aspects of FM (strong/weak points, assets/flaws, SWOT—*strengths, weaknesses, opportunities, threats*—analysis)

### Selection of articles

**Fig. 1:**
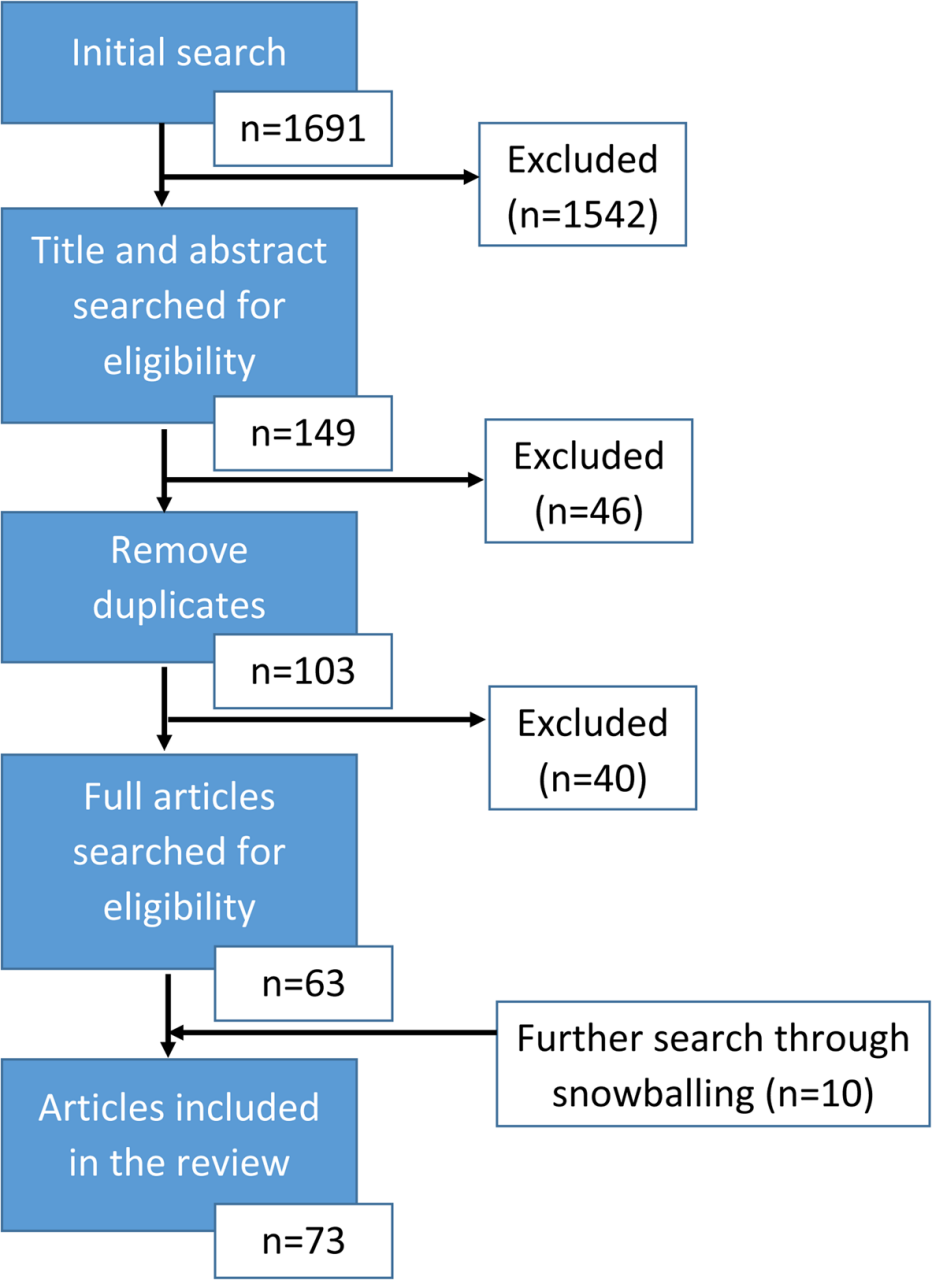
Flowchart of the article selection

Articles were initially selected using the title and abstract. Each of the team members focused on one of the search strings from Table 4. The first author reviewed all articles that were identified in the individual searches, taking out duplicates and creating the final list of selected articles. A total of 103 articles, with available abstracts, was obtained. Each of the researchers then received a list of approximately 20 articles to read the full text and reviewed if they met the inclusion and exclusion criteria. When the full text was not found or articles did not meet the defined criteria, they were excluded. Snowballing, retrieving relevant cited articles from the identified articles, was done until no more related articles were found [29]. Figure 1 shows the flow of article selection and the numbers that were in- and excluded [30].

### Data synthesis

Characteristics of the articles (such as authors, title, year of publication, journal, country focus, type of paper, aim, study population, methodology and key findings) were then extracted into a matrix. As this was a scoping review, no critical appraisal of the quality of the included articles was done [27]. The matrix was then used to analyse the characteristics of the included articles and interpret the key findings in order to answer the research questions.

### Identification of knowledge gaps

Key findings of these articles were compiled into a narrative which was presented in Gauteng, South Africa, April 2018, where a 3-day Primafamed workshop took place, with support of Stellenbosch University and Ariadne Labs. The Primafamed workshop also included the researchers from the other scoping reviews. Altogether, 15 participants came together to look at the findings of the scoping review and to validate the scoping review process. The knowledge gaps exposed by the scoping review were discussed. Following this meeting, the researchers completed the analysis and collaboratively wrote this article.

### Limitations of the methods

Only articles written in English were accepted. This limited the search mainly to articles from Anglophone Africa. Any articles from Francophone Africa and countries with other languages such as Portuguese, Arabic and Amharic were not included; therefore, information related to the development of FM in these countries was limited to English articles. The search focused on peer-reviewed articles from academic journals. Grey literature such as reports from ministries of health, non-governmental organizations and PhD theses were not included; therefore, there may have been additional evidence pertinent to the scoping review that was missed.

## Results

**Fig. 2:**
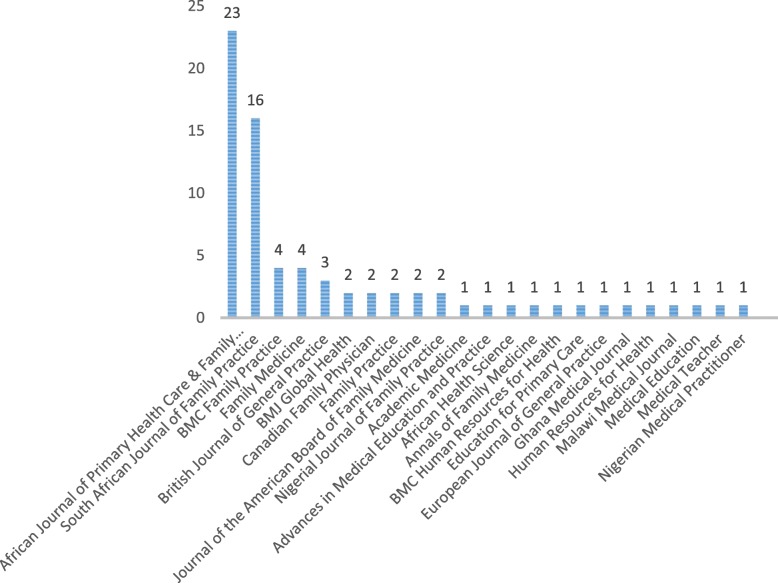
Number of publications per journal

Seventy-three articles were included from different journals (Table 7). The majority of articles were published in the African Journal of Primary Health Care and Family Medicine (32%) and the South African Family Practice journal (22%). Eighty-two percent were published in journals related to FM, 7% in journals related to education, 3% in journals related to human resources for health, 3% in global health journals and 5% in general medical journals. Thirty-eight percent of the articles were published in global journals, 33% in African journals and 29% in national journals (Fig. 2).

**Table 7:** Included studies in the scoping review

	Authors	Title	Publ. year	Journal	Geograph. focus	Type of paper	Methodology
1	Akoojee and Mash	Reaching national consensus on the core clinical skill outcomes for family medicine postgraduate training programmes in South Africa	2017	Afr J of PHC and FM	South Africa	Original research article	Delphi study
2	Arya Neil et al.	Family medicine around the world: overview by region. The Besrour Papers: a series on the state of family medicine in the world	2017	Can Family Physician	Global	Review	Scoping review
3	Arya Neil et al.	Developing family practice to respond to global health challenges	2017	Can Family Physician	Global	Review	Scoping review
4	Besigye and Namatovu	Scaling up Family Medicine in Uganda	2014	Afr J of PHC and FM	Uganda	Conference report	Not applicable (na)
5	Besigye et al.	Conference report: Undergraduate family medicine and primary care training in Sub-Saharan Africa: Reflections of the PRIMAFAMED network	2017	Afr J of PHC and FM	SSA	Conference report	na
6	Chege et al.	Evolution of Family Medicine in Kenya (1990s to date): a case study	2016	South Afr Fam Pract	Kenya	Review	Qualitative study
7	Couper and Mash	Obtaining consensus on core clinical skills for training in family medicine	2008	South Afr Fam Pract	South Africa	Original research article	Quantitative Survey
8	Couper and Hugo	Thoughts on the state of family medicine in South Africa	2008	Afr J of PHC and FM	SSA	Personal reflection	na
9	Couper et al.	Outcomes for family medicine postgraduate training in South Africa	2014	South Afr Fam Pract	South Africa	Personal reflection	na
10	De Maeseneer	Twenty years of Primafamed Network in Africa: Looking back at the future	2017	Afr J of PHC and FM	SSA	Editorial	na
11	De Maeseneer	Primary health care in Africa: now more than ever!	2009	Afr J of PHC and FM	SSA	Commentary	na
12	De Maeseneer	Scaling up Family Medicine and Primary Health Care in Africa: Statement of the Primafamed network, Victoria Falls, Zimbabwe	2013	Afr J of PHC and FM	SSA	Conference report	na
13	De Maeseneer and Flinkenflögel	Primary health care in Africa: do family physicians fit in?	2010	Brit J of Gen Pract	SSA	Commentary	na
14	De Villiers	Family medicine for Africa	2009	South Afr Fam Pract	SSA	Editorial	na
15	De Villiers and De Villiers	The current status and future needs of education and training in Family Medicine and Primary Care in South Africa	2002	Medical Education	South Africa	Commentary	na
16	Downing	Family Medicine: A profession for the world’s upper and middle class?	2010	Afr J of PHC and FM	SSA	Conference proceeding	na
17	Downing	African Family Medicine	2008	J Am Board Fam Med	SSA	Letter to the editor	na
18	Enabulele and Enabulele	Awareness & perception of the specialty of family medicine among medical students in a Nigerian medical school	2017	Nig J of Fam Pract	Nigeria	Original research article	Quantitative Survey
19	Essuman	Perceptions of Medical Students About Family Medicine in Ghana	2013	Ghana Medical Journal	Ghana	Original research article	Quantitative Survey
20	Evensen et al.	Family Medicine in Ethiopia: Lessons from a Global Collaboration	2017	J Am Board Fam Med	Ethiopia	Personal reflection	na
21	Flinkenflögel, et al.	Family medicine training in sub-Saharan Africa: South-South cooperation in the Primafamed project as strategy for development	2014	Family Practice	SSA	Original research article	Qualitative study
22	Franey et al.	Emergence of family medicine in Ethiopia: an international collaborative education model	2016	Education for Primary Care	Ethiopia	Review	na
23	Gaede	Rural health and family medicine	2010	Afr J of PHC and FM	South Africa	Commentary	na
24	Goodyear-Smith	Sub-Saharan Africa fast-tracks towards family medicine	2014	Family Practice	SSA	Editorial	na
25	Gossa et al.	Key informants’ perspectives on development of family medicine training programmes in Ethiopia	2016	Advances in Med Educ and Practice	Ethiopia	Original research article	Qualitative study
26	Hellenberg and Gibbs	Developing family medicine in South Africa: A new and important step for medical education	2007	Medical Teacher	South Africa	Commentary/ review	na
27	Hellenberg et al.	Family Medicine in South Africa: where are we now and where do we want to be?	2005	Europ J of Gen Pract	South Africa	Review	Scoping review
28	Hugo	Family Medicine as specialist discipline: Roots in History	2007	South Afr Fam Pract	South Africa	Editorial	na
29	Inem et al.	What Constitutes The Domain of Family Medicine in West Africa	2004	Nigerian Medical Practitioner	West Africa	Original research article	Qualitative study
30	Larson et al.	Current status of Family Medicine faculty development in sub-Saharan Africa	2017	Family Medicine	SSA	Original research article	Qualitative study
31	Lawson and Essuman	Country profile on family medicine and primary health care in Ghana	2016	Afr J of PHC and FM	Ghana	Original research article	Qualitative study
32	Makasa, Nzala, Sanders	Developing family medicine in Zambia	2015	Afr J of PHC and FM	Zambia	Commentary	na
33	Makwero, Lutala and McDonald	Family medicine training and practice in Malawi: History, progress, and the anticipated role of the family physician in the Malawian health system	2017	Malawi Medical Journal	Malawi	Review	Scoping review
34	Mash	Family medicine is coming of age in sub-Saharan Africa	2008	South Afr Fam Pract	South Africa	Letter to the editor	na
35	Mash	Reflections on the development of family medicine in the Western Cape: a 15 year review	2011	South Afr Fam Pract	South Africa	Original research article	Qualitative study
36	Mash	The contribution of family medicine to African health systems	2016	Afr J of PHC and FM	SSA	Commentary	na
37	Mash	The definition of family medicine in sub-Saharan Africa	2008	South Afr Fam Pract	South/ East Africa	Guest editorial	na
38	Mash and Reid	Statement of consensus on Family Medicine in Africa	2010	Afr J of PHC and FM	SSA	Conference report	na
39	Mash and Von Pressentin.	Family medicine in South Africa: exploring future scenarios.	2017	South Afr Fam Pract	South Africa	conference report	na
40	Mash et al.	Exploring the key principles of Family Medicine in sub-Saharan Africa: international Delphi consensus process	2008	South Afr Fam Pract	SSA	Original research article	Delphi study
41	Mash et al.	Guiding the development of Family Medicine training in Africa through collaboration with the Medical Education Partnership Initiative	2014	Academic Medicine	SSA	Report	na
42	Mash et al.	The contribution of family physicians to district health services: a national position paper for South Africa	2015	South Afr Fam Pract	South Africa	Position paper	na
43	Mash et al.	Reflections on family medicine and primary healthcare in sub-Saharan Africa	2018	BMJ Global Health	SSA	Editorial	na
44	Mash, Malan and Von Pressentin	Strengthening primary health care through primary care doctors	2016	South Afr Fam Pract	South Africa	Report	na
45	Mbuka et al.	New family medicine residency training programme: Residents’ perspectives from the University of Botswana	2016	Afr J of PHC and FM	Botswana	Original research article	Quantitative Survey
46	Mohamed et al.	Scaling up family medicine training in Gezira, Sudan – a 2-year in-service master programme using modern information and communication technology: a survey study	2014	Human Resources for Health	Sudan	Original research article	Quantitative Survey
47	Monjok et al.	Rural Health and Family Medicine: an agenda for sub-Saharan Africa	2011	Afr J of PHC and FM	SSA	Correspondence	na
48	Moosa et al.	The views of key leaders in South Africa on implementation of family medicine: critical role in the district health system	2014	BMC Family Practice	South Africa	Original research article	Qualitative study
49	Moosa et al.	African leaders’ views on critical human resource issues for the implementation of family medicine in Africa	2014	BMC Hum Res for Health	SSA	Original research article	Qualitative study
50	Moosa, et al.	Understanding of family medicine in Africa: a qualitative study of leaders’ views	2013	Brit J of Gen Pract	SSA	Original research article	Qualitative study
51	Moosa, et al.	Emerging role of family medicine in South Africa	2018	BMJ Global Health	South Africa	Commentary	na
52	Ogundipe and Mash	Development of Family Medicine training in Botswana: Views of key stakeholders in Ngamiland	2015	Afr J of PHC and FM	Botswana	Original research article	Qualitative study
53	Parsons et al.	Potential for the specialty of Family Medicine in Botswana: A discussion paper	2012	Afr J of PHC and FM	Botswana	Discussion paper	na
54	Pasio, Mash and Naledi	Development of a family physician impact assessment tool in the district health system of the Western Cape Province, South Africa	2015	BMC Family Practice	South Africa	Original research article	Mixed methods
55	Philpott et al.	The dawn of family medicine in Ethiopia	2014	Family Medicine	Ethiopia	Lesson from the field	na
56	Pressentin et al.	The perceived impact of family physicians on the district health system in South Africa : a cross-sectional survey	2018	BMC Family Practice	South Africa	Original research article	Quantitative Survey
57	Pressentin et al.	Examining the influence of family physician supply on district health system performance in South Africa: An ecological analysis of key health indicators	2017	Afr J of PHC and FM	South Africa	Original research article	Quantitative Survey
58	Pressentin et al.	The Influence of Family Physicians Within the South African District Health System: A Cross-Sectional Study	2018	Annals of Family Medicine	South Africa	Original research article	Quantitative Survey
59	Pressentin et al.	The bird’s-eye perspective: how do district health managers experience the impact of family physicians within the South African district health system? A qualitative study	2018	South Afr Fam Pract	South Africa	Original research article	Qualitative Survey
60	Reid	Names and roles for the generalist doctor in Africa. An email discussion between six family physicians	2010	Afr J of PHC and FM	SSA	Email discussion	na
61	Reid	The African family physician	2007	South Afr Fam Pract	SSA	Editorial/ Opinion	na
62	Reid	Community-oriented primary care: The missing link	2010	Afr J of PHC and FM	Global	Commentary	na
63	Robinson	Family medicine in Africa	2013	Brit J of Gen Pract	SSA	Opinion/ Commentary	na
64	Rouleau	Strengthening Primary Care Through Family Medicine Around the World: Collaborating Toward Promising Practices	2018	Family Medicine	Global	Review	Qualitative case studies
65	Setlhare	Reflections on Primary Health Care and Family Medicine in Botswana	2014	Afr J of PHC and FM	Botswana	Editorial	na
66	Setlhare, Mash and Tsima	The first National Family Medicine Conference in Botswana, May 2013	2013	Afr J of PHC and FM	Botswana	Conference report	na
67	Ssenyonga	Family Medicine may be helpful in improving health care delivery in sub-Saharan Africa.	2007	Afr Health Science	Uganda	Letter to the editor	na
68	Ssenyonga and Seremba	Family medicine's role in health care systems in Sub-Saharan Africa: Uganda as an example	2007	Family Medicine	Uganda	Regional Reports	na
69	Swanepoel et al.	Assessment of the impact of family physicians in the district health system of the Western Cape, South Africa	2014	Afr J of PHC and FM	South Africa	Original research article	Qualitative study
70	Tanko et al.	Awareness of family medicine discipline among clinical medical students of Bayero University, Kano, Nigeria	2017	South Afr Fam Pract	Nigeria	Original research article	Quantitative Survey
71	Udonwa, Ariba and Yohanna	Family Medicine in West Africa: progress, milestones, and challenges so far in Nigeria (1980 – 2010).	2011	Nig J of Fam Pract	West Africa	Review	Scoping review
72	Voort et al.	What challenges hamper Kenyan family physicians in pursuing their family medicine mandate? A qualitative study among family physicians and their colleagues	2012	BMC Family Practice	Kenya	Original research article	Qualitative study
73	Yakubu et al.	A qualitative study of young Nigerian family physicians’ views of their specialty	2017	South Afr Fam Pract	Nigeria	Original research article	Qualitative study

**Fig. 3:**
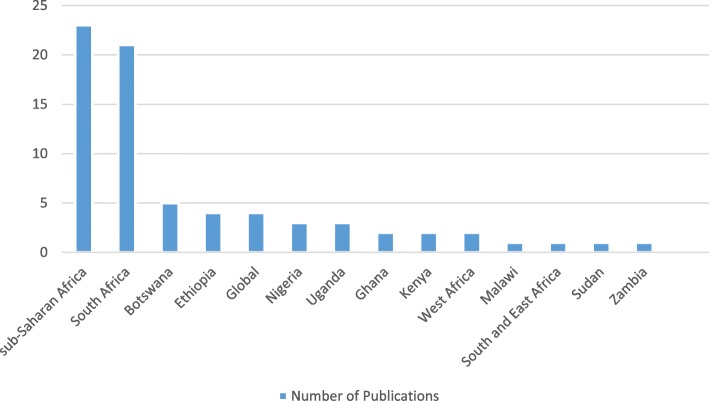
Geographical focus of the articles

Twenty-three articles (32%) focused on SSA and 21 (29%) specifically on South Africa. The remaining articles focused on African regions or specific countries (Fig. 3).

**Fig. 4:**
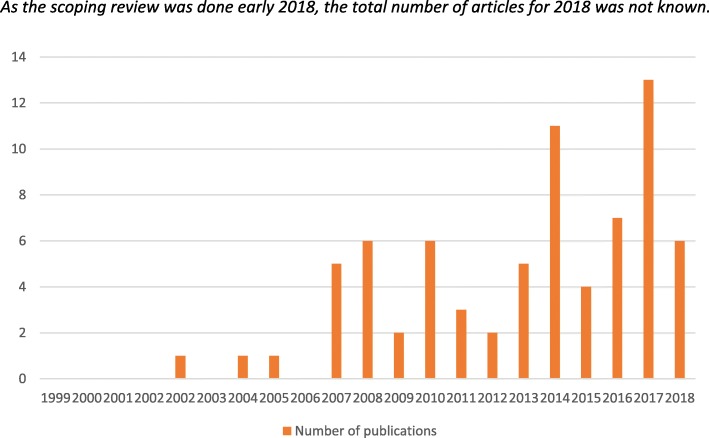
Number of publications per year. As the scoping review was done early 2018, the total number of articles for 2018 was not known

There was a clear increase of publications over the years, with a peak in 2017 (Fig. 4).

Of the 73 articles, 28 (36%) were original research articles. Out of these, 16 used qualitative research methods, 9 used quantitative surveys, two were Delphi studies and one was a mixed methods study. Five (7%) out of the 73 articles were literature review articles. The remaining 28 (44%) were a mix of commentaries, editorials, conference reports, position papers and personal reflection.

Experts acknowledged that “[Family Medicine] is limited by the lack of a regional definition. Governments, health departments and academic institutions would benefit from a clearer understanding of Family Medicine in an African context.” [11]. Therefore, in 2009 FPs and other stakeholders from all over SSA came together at the Rustenburg conference and a statement of consensus on FM in Africa was agreed upon (Table 8) [11].

**Table 8:** Rustenburg statement of consensus on family medicine in Africa [11]

1. “In an African context, the family physician is a clinical leader and consultant in the primary health care team, ensuring primary, continuing, comprehensive, holistic and personalised care of high quality to individuals, families and communities. 2. The family physician in Africa operates according to the principles of comprehensive person-centred care, with a family and community orientation, responding to undifferentiated illness and acting as a consultant to the primary health care team. 3. The role of the family physician in Africa involves a comprehensive set of skills adapted to the circumstances, local needs, available resources, facilities and the competency and limitations of the practitioner. 4. The family physician has a commitment and responsibility to a defined population to whom they are accountable through its representative structure. 5. The family physician's role requires close collaboration and teamwork with other members of the primary health care team, especially in the light of specific challenges, such as the insufficient numbers of health care workers. 6. The limited human, financial and material resources which exist necessitate skills appropriate to the situation. The family physician’s responsibility as consultant and gate-keeper encompasses the economic, effective and efficient use of available resources (human, financial and informational), as well as the ability to prioritize. 7. The family physician is also a life-long scholar, which includes a commitment to life-long learning, research and audit, and a responsibility for the continuing education of the primary health care team and community. 8. The family physician is an interdisciplinary player, with a pivotal role in the coordination of the primary health care team, including leadership in clinical governance and patient referrals. 9. Cultural competency in relation to language, gender, traditions and religious beliefs: is an essential attribute. 10. The family physician must play an advocacy role, both through daily example and through their institutions, by actively identifying with, and advocating for, the poor and marginalized. 11. The family physician should generate social and managerial accountability and transparency in terms of effective and efficient health care delivery. 12. Family physicians have a responsibility for health resource and service management based on their clinical understanding and should have direct access to District Health Management Teams. 13. The family physician may focus on various areas of special interest at different times in their career. At the same time, they must remain competent across a broad scope of practice as a generalist.” [11]	

### Key question 1: What are the different ways in which family medicine has been implemented in sub-Saharan Africa?

The implementation of FM was usually reflected in the way the postgraduate (PG) training of the discipline is delivered. Therefore, next to the actual implementation, some aspects of the delivery of the training will be provided.

The first developments in FM in SSA took place in South Africa and Nigeria, later East Africa and Ghana. Following this, most Southern African countries also introduced the discipline. There is no single model for FM. Each country had a unique set of circumstances that informed the most appropriate path for the development of FM, as shown in Table 9 [7, 17, 18].

**Table 9:** Implementation of family medicine in different African countries

Country	Development of FM	Focus of care/roles of FP	Positioning in the health system	References
South Africa	In 1968 University of Pretoria started with PG training, followed by the 7 other health sciences faculties. In 1997 FaMEC (Family Medicine Educational Consortium) was developed for standardization of the training and examination. In 2007 the government officially acknowledged the specialty. Presently there are 9 training programmes in place.	The 6 key roles as shown in Figure 5: Care provider, consultant, clinical trainer, capacity builder, leader of clinical governance, champion of COPC. Providing comprehensive care (preventative, curative, rehabilitative and palliative care)	At all levels of care; in primary, secondary and tertiary care settings. The FP functions at the district level, in district clinical specialist teams, at district hospitals and in health centres or sub-districts with multiple clinics. In rural as well as urban areas. FPs are also working in private general practice, non-public institutions.	[18, 32–38]
Nigeria	In 1970 General Medical Practice training started, in 1985 the first graduates entered the Nigerian health system. In 2004 the name changed to Family Medicine.	General physician, surgeon, obstetrician, gynaecologist and as community physicians	At all levels of care; in primary, secondary and tertiary care setting. In various settings such as military, universities, health centres, oil and other service industries, missionary hospitals, local governments, private practices and academia. In rural and urban district hospitals	[11, 12, 39–41]
Ghana	In 1991 FM extended from Nigeria to Ghana, where in 1999 the first residency programme officially started. In 2016 36 family physicians had been trained.	General physician, surgeon obstetrician, gynaecologist and as community physicians	In government health care facilities, mainly at the district hospital level. Around 15% in the private, military and quasi-government health facilities. In rural and urban areas	[42, 43]
Kenya	In 1998 first discussion took place with policymakers and subsequently a curriculum was developed. In 2005, the first trainees started the programme. By 2017, 29 Kenyan trained FPs had been deployed in the districts. Presently there are now 5 training programmes in country.	District health care with both inpatient and outpatient care, outreach to the community and emergency surgery and obstetrical skills	At all levels of care; in tertiary, secondary and primary care settings. The FP functions in the district health services, which includes clinics and district hospitals and extends to rural as well as urban areas	[6, 34, 44–46]
Sudan	Two year training programme started in 2010 with strong support from the government (the Gezira Family Medicine Program). In 2012, 207 FPs graduated from the first batch.	Lead the PHC team within the catchment area, comprehensive and community-oriented focus of care	Rural community health centres	[47, 48]
Uganda	Family Medicine was recognized by the government at its inception in 1989. In 2005 a national plan to train 1 FP per 75,000 inhabitants was conceived, but by 2013 only 20 out of these 400 were trained. This plan was revised and scaled up to train 600 FPs by 2025.	They are placed in roles as hospital directors and heads of community health departments, as well as clinicians caring for both in- and out-patients. Some head health districts providing leadership to district health teams	The Ministry of Health has positions for family physicians in national and regional referral hospitals and district hospitals, urban and rural	[13, 49]
Malawi	Discussion started in 2001, undergraduate FM clerkship started in 2011 and postgraduate FM training in 2015.	Competencies specific to the Malawian context; care provider, consultant, clinical leader and manager, community-oriented primary care leader, mentor and clinical teacher (including support of front-line primary care workers), researcher	District-level physicians and in primary health care teams, urban and rural	[50]
Botswana	In 2008 the Botswana Health Professions Council added FM to the list of registered specialties (they were trained in South Africa). The first school of medicine started in 2009 and FM training started in 2011. The first graduates have been employed in the health system.	Generalist doctors who can function within primary hospitals and lead primary care to transform quality and access to health care	Primary and secondary hospitals with outreach to the PHC platform	[51–55]
Ethiopia	Since 2001 the programme has been developed and in 2016 the first FPs graduated.	Generalists with internal medicine, pediatrics, surgery, psychiatry, emergency medicine, obstetrics and gynaecology, community medicine and public health competencies in order to lead a primary health care team in a local health care system	In well-equipped PHC facilities and district hospitals	[56–60]
Zambia	The FM training programme scheduled to start in 2015, officially started in 2018.	Holistic clinical and preventive care, healthcare management, research and clinical leadership	In rural, remote and underserved community-based settings within district health services	[61]
Rwanda	In 2008 the training programme started, but in 2010 the vision of the Ministry of Health changed, therefore ending the programme. The 9 graduated FPs have been taken up in the health system.	Presently none	Presently none	[18]
Mali	In 2017, a Master programme was being implemented.	No further information available	No further information available	[4, 60]
Somaliland	In 2017 a Master programme was being implemented.	No further information available	No further information available	[4]

Most postgraduate FM training programmes in SSA were inspired by the development of FM in Western Europe and North America. The literature, however, recognizes that the design of FM in HIC may not be applicable to SSA, as the pattern and distribution of diseases, shortages of healthcare workers and the rural location of the population are quite different [49]. FM training in SSA includes extended procedural skills especially for life-threatening medical, obstetric and surgical conditions in low-resource settings [32, 49].

Development of FM, with adaptation to local contexts, has taken place in many SSA countries, albeit in different ways and stages of development, as shown in Table 9. The Primafamed network showed that between 2008 and 2010, the developmental stage of FM training and the acknowledgement of the discipline in the different HS improved substantially for each of the participating universities [18].

In a survey on understanding FM in SSA, some key leaders saw FM as a specialized PHC physician. However, most saw African FPs mainly as hospital specialists, a combination of the four major clinical specialties or as stepping stones to later specialization, rather than a positive career option in its own right [5].

### Key question 2: What evidence exists for the effectiveness and impact of family medicine in sub-Saharan Africa?

FM in SSA ought to improve health outcomes, reduce costs, provide skilled leadership for PHC teams and improve the recruitment, retention and distribution of generalist physicians [57].

There is a clear perception among co-workers that FPs in South Africa are making an impact on quality of care and population health status [62]. In instances where FPs have functioned well, the PHC team has begun to function more coherently [62]. Reflections from district managers suggest that FPs make a significant impact on the quality of clinical processes and health system performance [63]. FPs have the potential to develop a sense of responsibility for specific communities and to connect higher management principles with local community needs. They also have been able to broaden the scope of practice as they received a comprehensive training, covering biomedical, psychological and social issues. As a result of the improved quality and scope of practice, FPs may have impacted on referral rates and enabled more patients to be managed in the district, saving money at other levels [31]. This also saved patients time and money, as previously people would have had to travel to a referral hospital [39].

Student perceptions in different countries also seem to be positive. Nigerian medical students believed FM was relevant as a specialty in the healthcare system, although most students preferred another discipline for their career choice [42]. The majority of first year undergraduate students at the University of Ghana perceived FPs to be capable of providing total health care for 85–95% of patients and also to reduce overall costs of care [44].

**Fig. 5:**
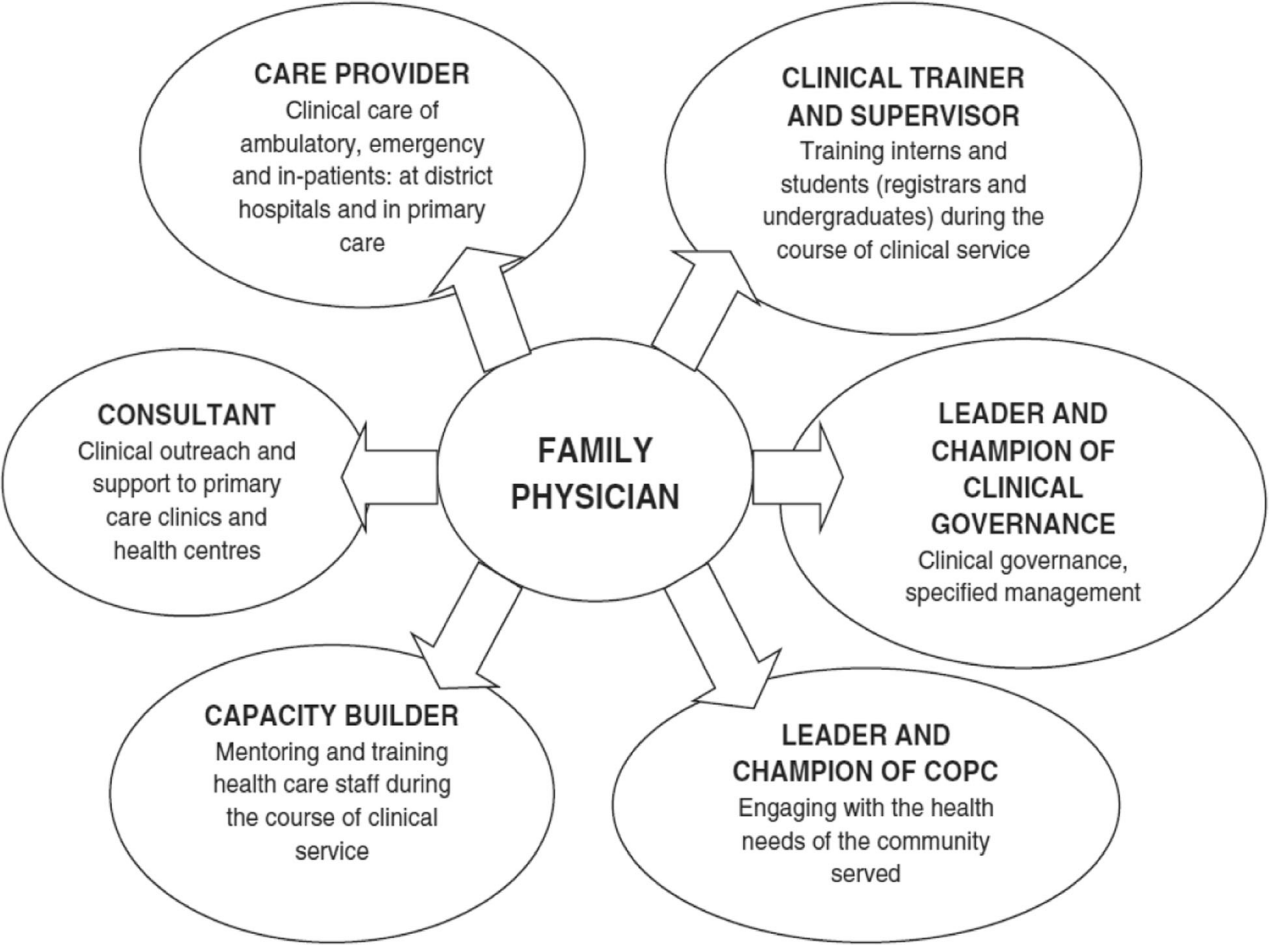
Roles and competencies expected of a family physician in South Africa [31]

A FP impact evaluation tool was developed in South Africa and used in a national survey. This survey questioned managers, doctors, nurses and other health professionals working with FPs. Family physicians were perceived to have a high impact in their roles as clinicians, consultants, clinical trainers, leaders of clinical governance and champions of community-oriented primary care and a moderate impact as capacity builders of the health care team. This impact was perceived to be significantly more than medical doctors (without specialty training) across all six key roles [64]. These key roles for FPs were agreed upon in South Africa as shown in Fig. 5 [39, 64]. This higher perceived impact was found in district hospitals and primary care facilities as well as urban and rural areas. An additional study found evidence that FPs were making a significant impact at district hospitals, particularly in child health care [65]. Surprisingly, this same study found that community health centers without FPs had better continuity and coordination of care, although this might be due to the confounder that FPs were placed at larger centers with a higher workload [65]. There was no correlation between FP supply and routinely collected district health indicators as numbers of FPs were still very small (0.3 per 10,000 population in the public sector) [65, 66].

### Key question 3: What is known about the strengths and weaknesses of family medicine as part of health systems in sub-Saharan Africa?

A huge challenge is that FM is still fairly unknown, and due to low numbers, there is low visibility. In many countries FM is still in its early stages of development and there is little opportunity to assess strengths and weaknesses at scale [67]. However, policymakers, funders and other disciplines require more evidence to shift to a more positive attitude [5]. Roles and responsibilities of FPs are not always clear, neither is their exact place in HS, leading to difficulties with incorporating the discipline into health policy [5].

A continuous challenge around the continent is training sufficient FPs to show a significant impact on health outcomes [57]. Key stakeholders can sustain or sink the development of a programme and support is context dependent. While in several countries, such as South Africa, Nigeria, Kenya, Ethiopia and Ghana, it appears that decision makers find value in FM, other stakeholders (Table 10) have different perspectives on the discipline. In a number of other countries, such as Rwanda and Tanzania, support for its development is almost absent [4].

**Table 10:** The ambiguity of how family medicine is experienced in Kenya [6, 44]

*The general population* experiences family physicians as consultants working in district hospitals where they run outpatient clinics, conduct teaching ward rounds and perform significant major emergency general and reproductive health surgery *Policymakers* report these generalists as ‘the wonder doctors’ who demonstrate unique all-round competencies *Other specialists* tend to look down on FPs and see them as intruders to their specialties *Other academics* find it hard to differentiate between family medicine and the different disciplines	

A 2010 study among FM educators revealed many challenges such as a need for more FP trainers, funding, resources, career opportunities, buy-in from hospital-based specialists and acceptance of FM as an essential discipline by authorities to train the required critical mass [18, 68].

Within the discipline in SSA, there is some debate on whether FM should move away from a hospital-based focus towards a more primary and community-based focus (Table 11) [69, 70].

**Table 11:** Thoughts on the state of family medicine in South Africa, strengths and concerns by Couper et al. [69]

“We (family physicians) need to be very clear that we are different from other specialties, and not try to be the same. We think that some of our problems derive from the fact that we try to be the same as other specialties and to be seen in the same way, instead of making it very clear that we are completely different, because primary care is different from any other specialty; because our role is in the community, and not in the hospital like other specialists; because our focus is on all patients and not types of diseases or specific groups of patients; and because our approach is holistic, rather than specific. We are generalists who need to coordinate patient care in balance with specialists, who each have their own unique way of making clinical decisions. We need to be experts in health, and to say to our patients that their illnesses are but one part of them as whole people, while the specialist is an expert in saying which sicknesses they do or do not have, in a narrow field.” “We are extremely worried by reports of family physician specialists who consider themselves to be too important to see patients with so-called minor ailments. We are deeply disappointed to hear students reporting on family physician colleagues saying: “I am a specialist family physician” with great pride, as they strut around and do not see the patients that the other doctors and nurses see, because they are specialists. We feel pain when we hear that our colleagues will not carry out the normal first contact calls, but want instead to perform “consultant calls”, where they sit at home and are only called out on the odd occasion, while still being paid the full amount for overtime. Is that what being a specialist really means? Are we selling ourselves out? This is definitely not the way to gain the respect of our colleagues, the public, or the powers that be that run the health service. We do not think it is the way to gain self-respect either.” [65]	

Several strengths of the discipline have been identified. The creation of specialist FP posts within the public sectors of countries in Western and Southern Africa has established a career pathway with the same salary scale as other specialists in the academic teaching hospitals. This has enabled FM to attract good candidates and to start to transform the perception that only people without academic ability or ambition are attracted to careers in district health services. The district health services have seen FPs make a strong contribution in the area of clinical governance [39]. Key leaders saw the capacity to provide training, mentorship, supervision and leadership as some of the strengths (Table 12) [5].

**Table 12:** Benefits and concerns in relation to Family Medicine mentioned by key leaders [5]

Benefits	Concerns
• A clinically skilled generalist all-rounder at the district hospital • Mentoring team-based care in the community • A strong leadership role in the district health system • Developing comprehensive holistic practice of medicine • Focus on community care, such as community-oriented primary care (COPC)	• Family medicine is unknown or poorly understood • Poor recognition, visibility and role clarity • Struggling with policy ambivalence and needs advocacy • Slow pace of FPs being trained and low numbers of FPs placed into the health system

### Key question 4: Where are family physicians deployed in sub-Saharan African health systems?

FPs are seen to be a communicator, collaborator and consultant strengthening care delivery. They are placed between non-physician primary care providers (such as nurses in the health centres) and specialist physicians at higher levels of care. The FP’s niche is often said to be in the community, but they are usually placed at other levels of care due to low numbers of human resources for health [6]. This tension between concept and reality is a challenge for the identity of the discipline. FPs are employed at all levels of care, depending on the country’s health system, available other human resources and the local needs, in primary, secondary and even tertiary care [36].

Key leaders saw FPs as “all-round specialists” (or expert-generalists) at smaller hospitals, in the absence of other specialists [5]. In most SSA countries, FPs are deployed in the framework of the district health system, which includes primary care facilities and district hospitals in both rural and urban areas (Table 9) [39]. The skills gap at district hospitals, often in rural or remote areas, was a compelling argument for the inclusion of FPs in the South African health system as these hospitals were not likely to sustain or attract other specialists [39].

### Key question 5: What roles do family physicians play in sub-Saharan African health systems?

The 2009 Rustenburg consensus (Table 8) related the roles of FPs in SSA to “a comprehensive set of skills adapted to the circumstances, local needs, available resources, facilities and the competency and limitations of the practitioner” [11]. Due to the different interpretations of the practice, some argue that “searching for a role-based common definition is ultimately insufficient” [8].

The model that emerged in South Africa required the FP to work at district hospital and in PHC, with the key roles as shown in Fig. 5. As care providers, they needed to be able to manage the majority of patients presenting to the district hospital and health centres, while as consultants to the inter-professional teams they actually saw the more complex medical problems. As capacity builders, they delegated tasks and responsibilities, while giving support and training to other members of the team. As clinical trainers, they provided training and supervision to the resident FPs, interns and medical students. As leaders of clinical governance, they led the teams in improving quality of care and patient safety, while as champions in COPC they supported the PHC teams in engaging with local communities to improve population health [15, 39, 71].

In West Africa, FM roles included PHC that could be in the home or primary care facility, with a focus on family-oriented primary care as well as in secondary or tertiary care hospitals [40, 72]. FPs were also placed in roles as clinical managers and medical superintendents throughout SSA, where they would manage systems, finances, schedules and patients and work as “agents of change” [6, 13, 43].

In many countries, the definition of roles is still “work in progress” [48, 50]. In Ethiopia for example, roles and responsibilities have not yet been clearly defined. FPs are expected to be care providers in the primary and secondary care level and act as health managers and team leaders [57].

Despite the overall intuitive consensus of FM being comprehensive and holistic, there tend to be two major directions. One direction, due to low human resources and significant skills gaps, sees the need to support district hospitals with outreach from there to PHC and the community [8]. FPs, particularly in rural areas, need to have additional training in obstetrics, surgery, otolaryngology, ophthalmology and child health and mental health as “rural family medicine training in Africa should continue to include skills necessary for secondary health care at the district level until all developmental indices, including medical manpower, has engulfed the vast rural communities in these countries” [70]. In the other direction, FPs are seen as supporting and being part of PHC teams in the community, with a focus on COPC [72]. In South Africa, the national position paper embraces the need to train and deploy FPs in both ways throughout the district health services and not to choose between these options [73].

During a 2016 workshop on exploring future scenarios of FM in the South African health care system, a group of 40 FPs came to three possible scenarios (Table 13), all depending on the direction policymakers would decide upon. They identified the need for increased advocacy for the discipline, especially in rural areas, and to increase research evidence of the contribution of FM to the health system [15].

**Table 13:** Possible future scenarios of family medicine in South Afric a[15].

In the *most positive scenario* the National Health Insurance is implemented with full focus on UHC, with PHC and family medicine at the heart of the health system, where FPs work in multidisciplinary teams in the community and in district rural hospitals. In the *continuation of present scenario*, the system continues to struggle with resources and quality in the public sector, with family physicians not fully integrated in the health system and struggling with the low numbers, especially in rural and remote areas. In the *most pessimistic scenario*, the National Health Insurance system is poorly implemented and family physicians leave for the private sector or overseas, the Department of Health decides to focus on other areas and end the deployment of family physicians.	

## Discussion

The practice of FM varies from country to country, depending on the country’s health system, the presence of means and manpower, the needs of the community and the burden of disease. There are a number of unifying principles: socially accountable responsiveness to local needs, adaptation to the existing health care system and ongoing development of the competencies required to succeed in the six professional roles, always grounded in relationships of care. In this way, FM is evolving to suit the health needs of communities and countries [5, 7, 11].

Ongoing discussion has been happening within the discipline between theoretical models, sometimes derived from HIC, and the reality of practice in SSA. Even within countries, there can be quite a different scope of practice related to specific needs. For example, roles are different for FPs working in urban health centres versus rural district hospitals. FPs are providing clinical care, including emergency surgical, anaesthetic and obstetrical care where appropriate. At the same time, they have roles that are oriented towards the community, public health, clinical training, capacity building skills, clinical governance and sometimes even managerial responsibilities.

As regards to positioning in the health care system, FPs are mainly placed in the district hospital with outreach to health centres, PHC teams and communities [74]. At regional or tertiary hospitals, FPs have been working to triage patients for other specialists or to fill gaps where specialists are absent. This leads to variations in the job descriptions, despite the core competencies being the same. There is a tension between training for hospital-based care and primary care. Some researchers warned that when FPs are fully drawn into hospital-based specialist roles, it can undermine the holistic approach at the heart of medical generalism and therefore they argued to focus more on COPC [69]. FM is a discipline that is fluid, adjusting to the situation and developing over time. African health systems currently see the need for FPs at district hospitals more than in PHC and this may be because of the small numbers of FPs, a hospital-centric perspective, significant skills gap in district hospitals that other specialists are unlikely to address, and historic absence of such expertise in PHC teams. The discussion on deployment (and training) of FM at the primary, secondary and/ or tertiary level in the health systems may be transitionary and needs further exploration.

Brain drain and retention of physicians is a huge challenge in SSA and has weakened the physician workforce of many countries [75, 76]. Movement may be internal, to vertical disease-specific programmes, specialist care, government, urban areas and non-clinical work; and external, from central to South Africa and overseas [76–78]. There is a sense that FM may help to reduce this internal and external movement [57, 67], though clear evidence was not found to support this hypothesis.

In international literature, it is often stated that FPs are able to deal with 90% of the disease burden within their context [51]. However, evidence for this in the SSA setting was not found. Measuring the impact and effectiveness of the discipline will remain challenging. Low numbers of FPs and little research capacity to address such questions has limited the evidence available. Some initial evidence of impact has come from South Africa, but more is needed in SSA [67]. Before evidence can be demonstrated, there is a strong need for advocacy to get the discipline to the attention of policymakers and future health care leaders, in order to create the necessary critical mass of FPs in the health systems. Proof that high level commitment is possible has been shown in Sudan, where 2 years after implementation of the programme 207 FPs graduated and were deployed to health centres throughout the district, the majority of which were never served by a doctor before the programme [48].

UHC with equitable, high-quality care can be achieved through well-trained PHC workers including FPs, but sufficient numbers are essential [24]. Challenges include the lack of buy-in from hospital-based specialists and acceptance of FM by authorities [68]. Increased financial resources are needed. Initiatives such as shifting money from vertical disease-oriented programmes for capacity building and to strengthen FM and PHC should be pushed for [79].

There has been a move towards strengthening PHC in the region and to incorporate PHC and FM in undergraduate medical education. This may also lead to more openness among policymakers to consider including FM in health systems. This may lead to more exposure of medical students to PHC and a greater likelihood of them considering FM as a career [42, 80, 81].

Another key finding was the importance of collaboration and support between universities, both South-South and North-South as shown by the Primafamed network, in order to develop the discipline in SSA. Good communication and collaboration with policymakers and other key stakeholders was another important finding [8, 18, 45, 58]. Presently, FPs are most often not mentioned in policy documents in SSA [82]. Strong commitment from policymakers is pivotal to train sufficient numbers of FPs.

A study bias was that the researchers all had a strong background in FM in SSA and therefore their views were strongly shaped by their own situations. The researchers used a strict scientific approach while working on this scoping review, taking advantage of their knowledge of the field. Such insights also led to a better understanding of the complexity of the subject. Most research found in this review came from South Africa, fewer findings were from the other SSA countries. As mentioned earlier, another limitation of the study was the focus on English publications from peer-reviewed journals only. There has been some recent development of FM in Francophone Africa, such as DRC and West Africa; though in the literature we explored, little was written related to these countries. During the latest Primafamed meeting in Kampala (2019), expansion to Francophone Africa was extensively discussed. Collaboration and further research is necessary to strengthen FM development throughout the whole of SSA.

The main knowledge gap is the need for more evidence on the ways in which FM is implemented, particularly in countries outside of South Africa, and the need for more evidence on the effectiveness and impact of FM on health systems and outcomes. There is also a need to measure the cost-effectiveness of deploying FPs versus other types of health professionals. Research methods may need be tailored to the numbers of FPs. For example, case studies and more qualitative exploration of impact may be useful initially, while more quantitative observational studies may be useful when numbers are increased. Although the Lancet argued that FM “is so integral to the path towards the SDGs that reference [to it] in a goal or target would undermine its cross-cutting role” [3], more evidence is still crucial.

## Conclusions

FM is still evolving in SSA, and more than any other specialty, it is responsive to the specific needs of the populations it serves, organizational models and health system designs. Therefore, no single, clear answer to the different questions we posed came out of this scoping review. The findings were numerous and depended on the different settings in SSA. In most settings, FPs are placed in district hospitals and work from there with PHC teams. FM is continuously adapting to the changes in the HS, burden of disease and the local needs.

Evidence of effectiveness and impact is still limited as the discipline is reasonably young in SSA with low numbers of FPs. Opposition due to lack of understanding remains, but the positive perceptions of key stakeholders and the motivation of FPs, together with evidence from elsewhere, suggest that the discipline can fill a niche and potentially improve quality of care in SSA. Political will and support is pivotal and will enable the discipline to create the critical mass to place FM at the forefront, to reach UHC and contribute to the achievement of the SDGs in sub-Saharan Africa.

## Data Availability

The full list of articles used for this review can be found in Table 9.

## Abbreviations

DRC: Democratic Republic of Congo
COPC: Community-oriented primary care
HS: Health systems
FaMEC: Family Medicine Education Consortium
FM: Family medicine
FPs: Family physicians
GPs: General practitioners
HIC: High-income countries
LMIC: Low- and middle-income countries
MDs: Medical doctors
MeSH: Medical subject heading
NGOs: Non-governmental organizations
PHC: Primary health care
PHCFM: Primary Health Care and Family Medicine
PG: Postgraduate
SDGs: Sustainable Development Goals
SSA: Sub-Saharan Africa
SWOT: Strengths, weaknesses, opportunities, threats
UHC: Universal health coverage
WHO: World Health Organization

## Footnotes

1 FM in francophone Africa was not addressed in this reference
